# Exposure of Dural Venous Sinuses: A Review of Techniques and Description of a Single-piece Troughed Craniotomy

**DOI:** 10.7759/cureus.2184

**Published:** 2018-02-12

**Authors:** William E Gordon, L. Madison Michael II, Matthew A VanLandingham

**Affiliations:** 1 Neurosurgery, University of Tennessee Health Science Center; 2 Neurosurgery, Semmes-Murphey Clinic

**Keywords:** superior sagittal sinus, transverse sinus, craniotomy, parasagittal lesions, interhemispheric fissure, literature review

## Abstract

Intracranial lesions along the falx and tentorium often require exposure of a dural venous sinus. Craniotomies that cross a sinus should maximize exposure while minimizing the risk of sinus injury and provide a cosmetically appealing result with simple reconstruction techniques.

We describe the published techniques for exposing dural venous sinuses, and introduce a new technique for a single-piece craniotomy exposing the superior sagittal sinus or transverse sinus using drilled troughs.

A review of the literature was performed to identify articles detailing operative techniques for craniotomies over dural venous sinuses. Our troughed craniotomy for dural sinus exposure is described in detail as well as our experience using this technique in 82 consecutive cases from 2007-2015.

Five distinct techniques for exposure of the dural venous sinus were identified in the literature. In our series of patients undergoing a trough craniotomy, there were no sinus injuries despite a range of various locations and pathology along the sagittal and transverse sinuses. Our technique was found to be safe and simple to reconstruct compared to other techniques found in the literature.

A variety of different techniques for exposing the dural venous sinuses are available. A single-piece craniotomy using a trough technique is a safe means to achieve venous sinus exposure with minimal reconstruction required. Surgeons should consider this method when removing lesions adjacent to the falx or tentorium.

## Introduction

The approach to intracranial lesions adjacent to the major venous sinuses often necessitates complete sinus exposure. Likewise, visualization of deep midline intracranial lesions benefits from complete exposure of the superior sagittal sinus by substantially increasing the working angle [1]. Typically, the dura is densely adherent to the skull immediately over the venous sinuses, which poses a risk of sinus injury during removal of the overlying bone. Direct sinus injury or laceration of adjacent venous lakes and bridging veins can not only cause substantial blood loss but can also cause possible air embolism or venous infarct.

All techniques for performing a craniotomy over a venous sinus share the common goal of minimizing risk of injury to the underlying sinus. Secondarily, the craniotomy technique should be as simple as possible to maximize surgical efficiency and minimize reconstruction time and costs. We present our technique of a troughed craniotomy over the sinus in 82 consecutive patients as well as a review of existing techniques to expose the dural sinuses.

## Technical report

Literature search

A PubMed search was utilized to identify articles detailing unique operative techniques for craniotomies over dural venous sinuses. Search terms included: craniotomy, dural venous sinus, superior sagittal sinus, interhemispheric approach, trough craniotomy, and parasagittal craniotomy. Five unique techniques for exposure of the dural venous sinus were identified in the literature.

Author technique

**Figure 1:**
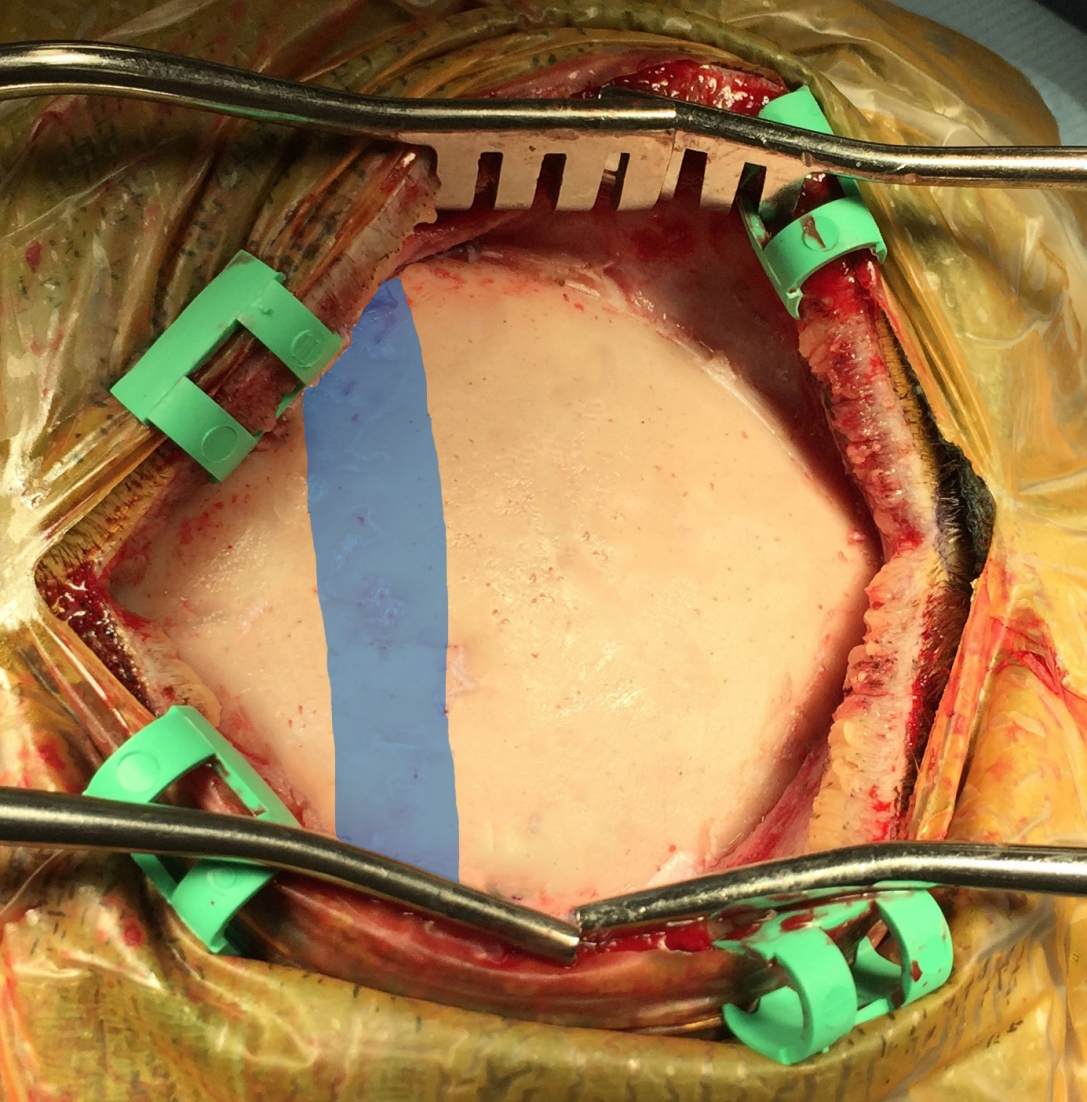
Exposure of the calvaria overlying the sagittal sinus.

**Figure 2:**
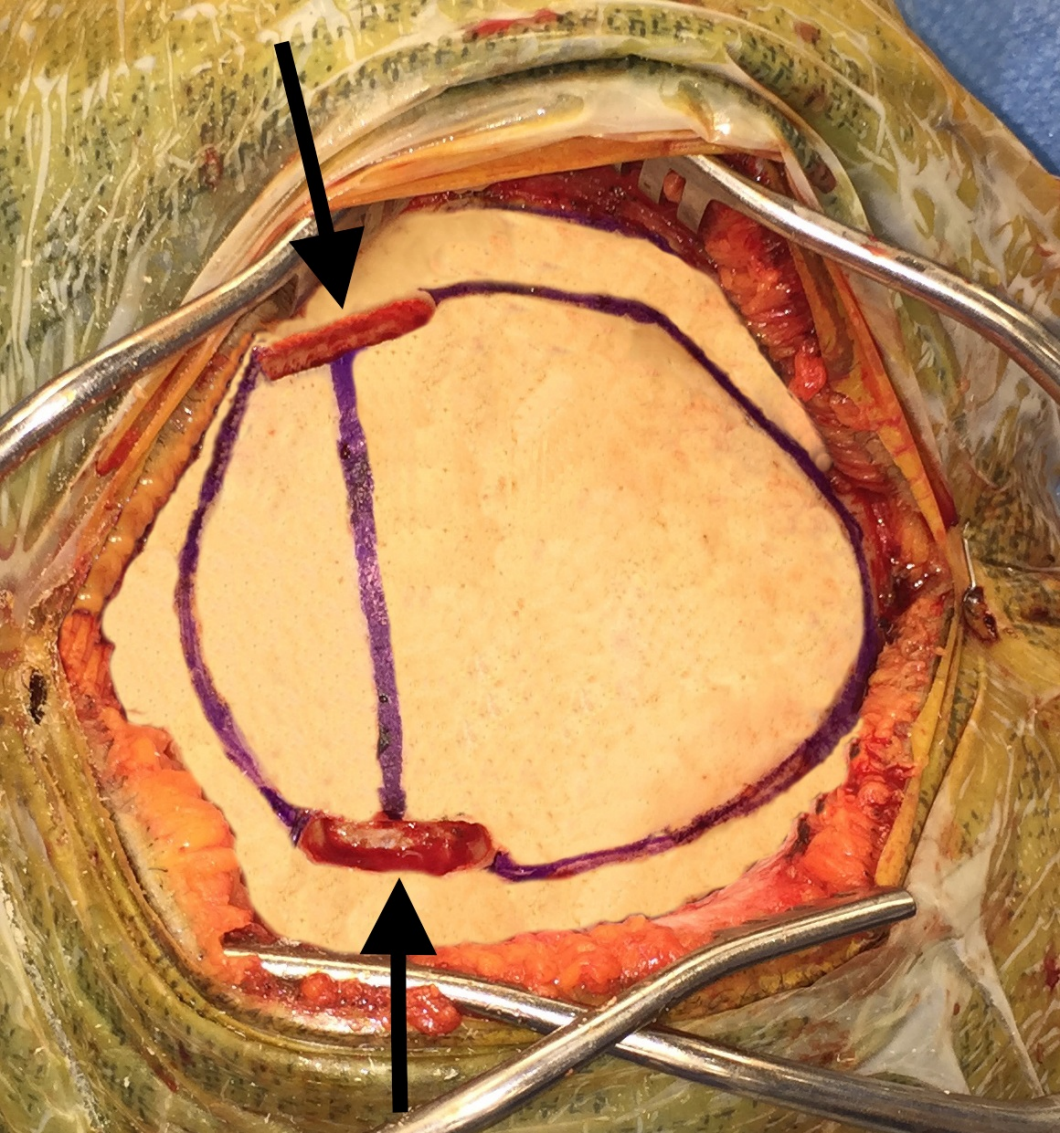
The Midas Rex 8MH17 bit is used to trough directly over the sinus, exposing the lateral edges.

**Figure 3:**
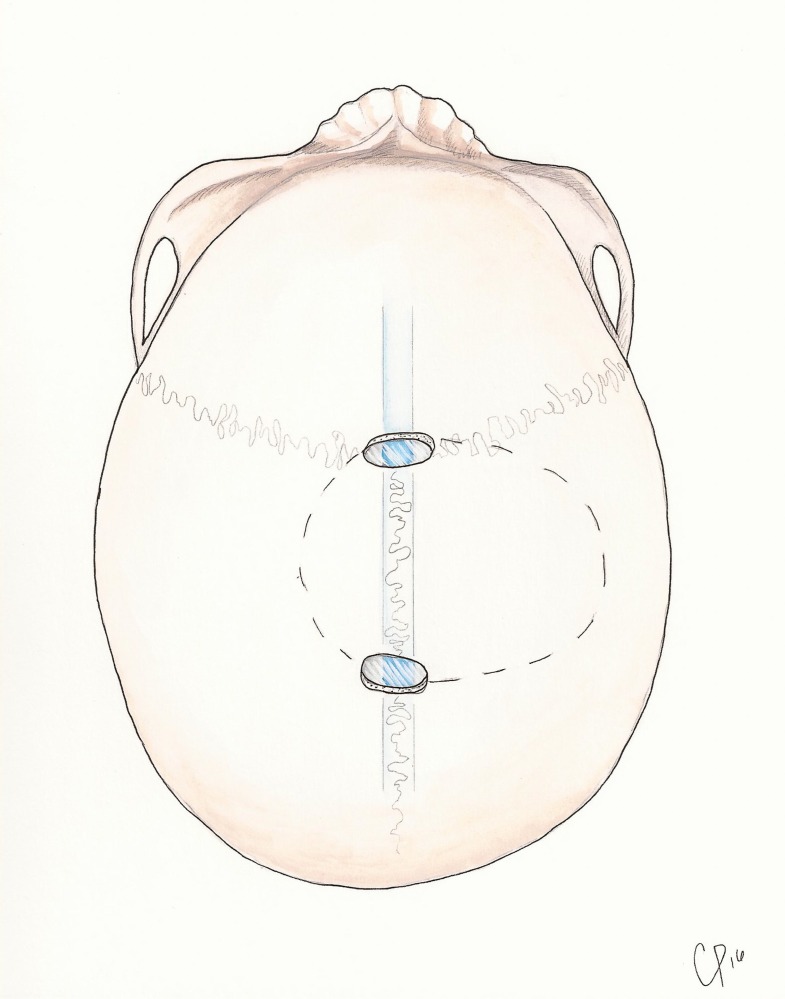
Illustrated steps of turning a one-piece trough craniotomy over the sagittal sinus.

**Figure 4:**
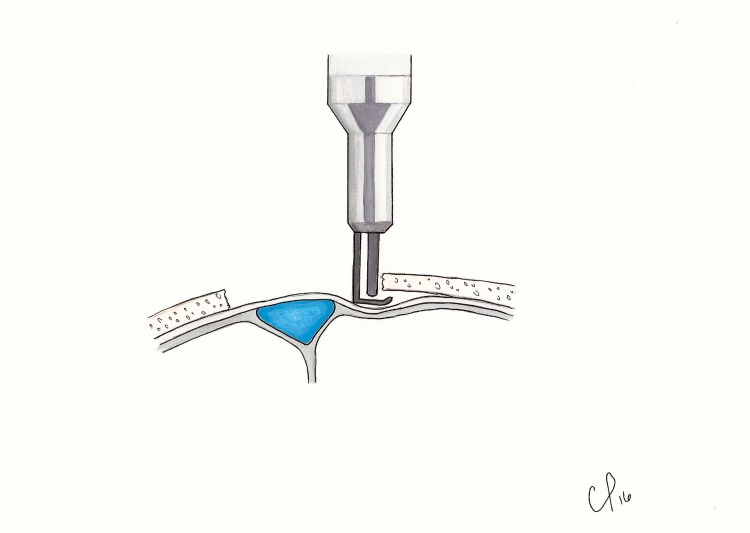
The footplate attachment is used to free the dura laterally from the trough.

**Figure 5:**
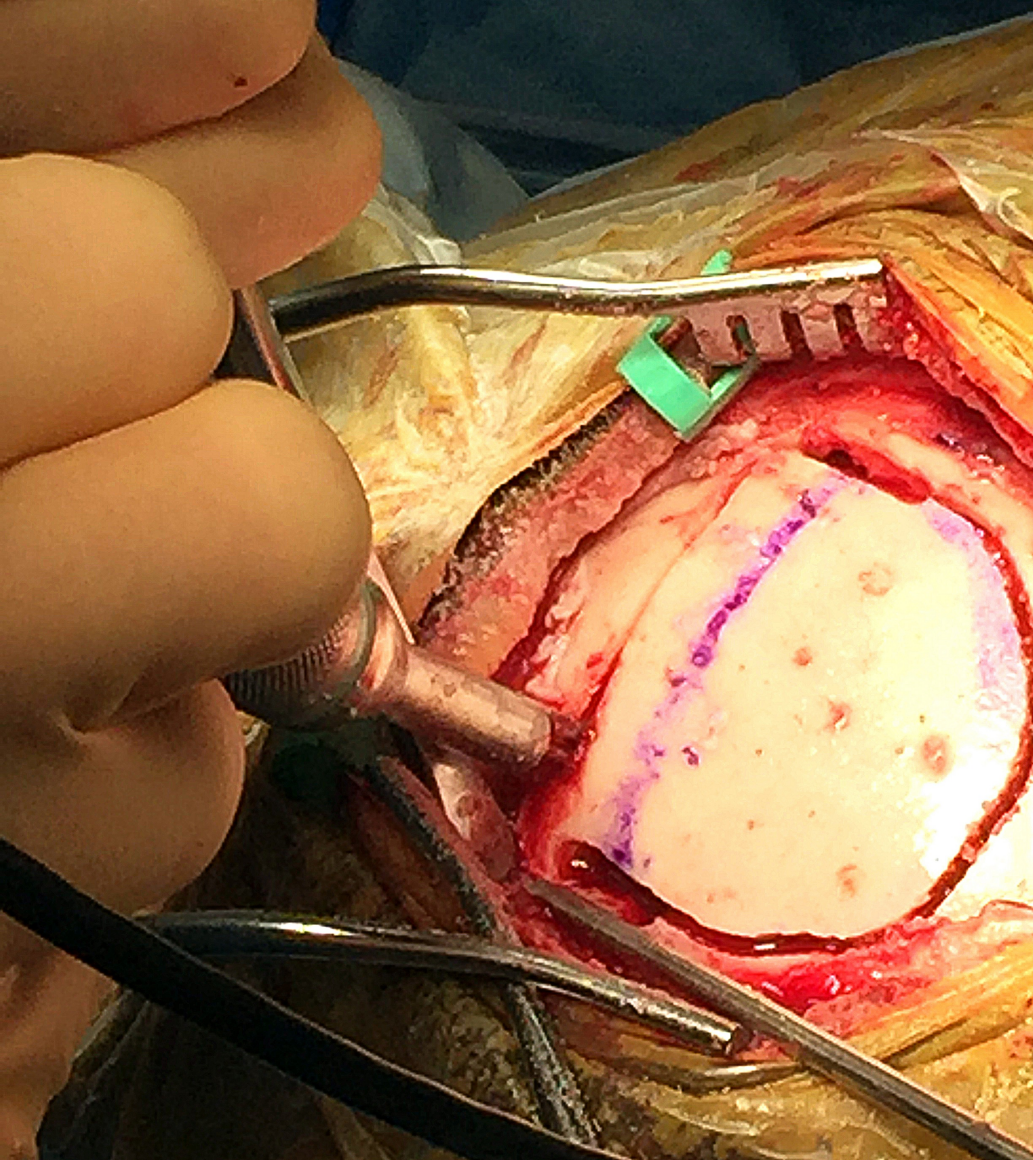
The B1 drill bit with footplate is used to complete the craniotomy.

**Figure 6:**
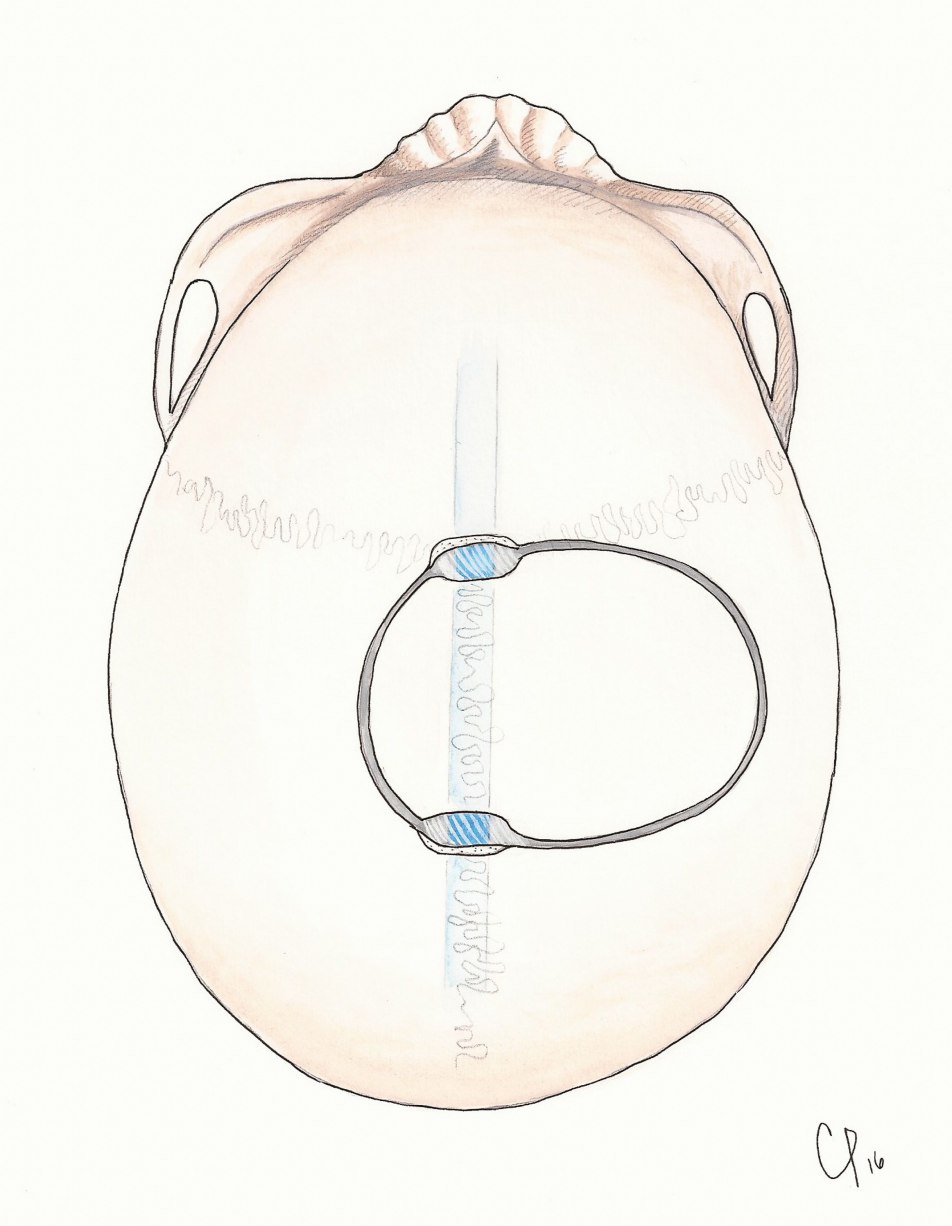
The craniotomy is directed away from the sinus.

**Figure 7:**
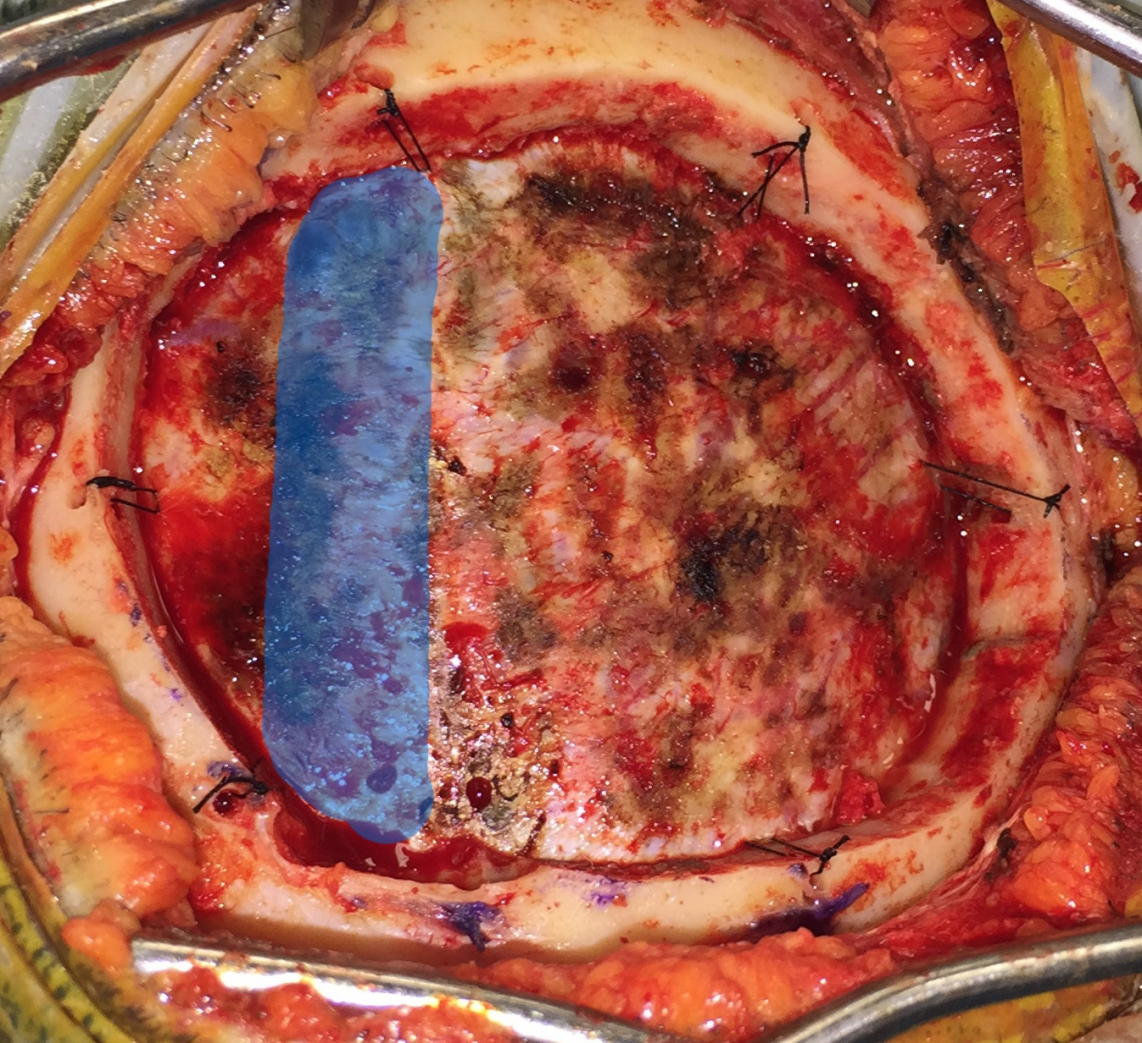
The bone flap is elevated from the side furthest from the sinus, while providing gentle counter force on the opposite side to prevent levering the bone flap into the sinus. Dural tack-up sutures are placed around the perimeter of the craniotomy. The location of the sagittal sinus is highlighted in blue.

For our technique, each patient underwent a preoperative MRI brain with and without contrast that was integrated into the Medtronic StealthStation Surgical Navigation System (Medtronic; Surgical Technologies, Neurosurgery; Louisville, CO, USA) in the operating room. The surgical site was then mapped out using the StealthStation, and the patient was positioned on the operative table accordingly. After exposure of the calvarium (Figure 1), in our series, a Medtronic Midas Rex 8MH17 drill (Medtronic Powered Surgical Solutions; Fort Worth, TX, USA) was used to make an anterior and posterior trough perpendicular to the sinus, although it should be noted that any similar atraumatic drill bit could be utilized. The tip of the 8MH17 drill bit is blunt with the cutting edges on the sides of the bit. Thus, atraumatic removal of bone can be achieved when using the bit perpendicular to dural venous sinuses. The trough cut is taken laterally away from the sinus until dura can be visualized on both sides (Figures 2-3). If there is any doubt regarding exposure of dura, further drilling away from the venous sinus is performed. The dura is then separated from the calvaria at the lateral aspects of the trough using the footplate attachment for the Midas Rex drill (Figure 4). The craniotomy is then completed using the Midas Rex B1 drill bit with footplate (Figures 5-6). The bone flap is gently elevated from the side furthest from the sinus first, using a Penfield instrument to dissect the dura off the inner table of the bone flap, while providing gentle counter force on the opposite side to prevent levering the bone flap into the sinus (Figure 7). In this way, the dura over the sinus can be stripped of the bone under direct visualization. Dural tack-up sutures are then placed around the perimeter of the craniotomy. The remainder of the operation proceeds as the pathology dictates.

Results

A literature review identified five distinct techniques for performing a craniotomy that crosses a venous sinus. The most frequently reported technique involves placement of a burr hole on either side of the sinus, freeing the underlying dura and venous sinus with a blunt instrument. The bone flap is elevated following this maneuver [2-5]. A variation on this technique places only one burr hole adjacent to the sinus with the contralateral burr hole placed at the lateral extent of the craniotomy [6]. Alternatively, a two-piece craniotomy has been described in which the first portion of the bone flap is elevated adjacent to the sinus, allowing the dura over the sinus to be freed from overlying bone under direct visualization before elevating the second portion of the bone flap over the sinus [7-8].^ ^Drilling directly over the sinus has also been described, either by placement of burr holes over the sinus [9-10] or use of an oscillating saw over the sinus [11].

In our series, the median patient age at the time of surgery was 60 years (range 18–85). Of the 82 patients, 48 (59%) were female and 34 (41%) were male. There were no intraoperative complications in our series. Specifically, there were no direct or indirect injuries to the venous sinuses. The median hospital stay was 4.3 days (range 1–25 days). The most common pathology addressed was meningioma (Table 1). The middle third of the sagittal sinus was exposed in the majority of craniotomies, although all sections of the sagittal and transverse sinuses were represented in our series (Table 2). Thirty-two (39%) of the tumors were right-sided and 28 (34%) were left-sided; 22 (27%) were bilateral.

**Table 1:** Pathology found in patients undergoing craniotomy

Pathology	Patients with Pathology (No.)	Patients with Pathology (%)
Meningioma	33	40.24%
Metastatic	14	17.07%
Glioblastoma	9	10.98%
Colloid cyst	3	3.66%
Schwannoma	2	2.44%
Oligodendroglioma	2	2.44%
Other	19	23.17%
Total	82	100%

**Table 2:** Location of craniotomy

Laterality	Patients (No.)	Patients (%)
Frontal	39	47.56%
Occipital	25	30.49%
Parietal	18	22.95%
Total	82	100%

## Discussion

The goals of any craniotomy should be to obtain safe and adequate exposure. It will allow an unhindered approach to the intracranial pathology resulting in the least amount of manipulation and traumatization of the brain. Performing a craniotomy that crosses a major venous sinus necessitates proper technique to avoid significant blood loss, air embolism, and venous stroke.

Numerous methods have been described for sinus exposure. Burr holes placed on either side of the sinus require blindly freeing the dura between, risking direct laceration, and injury of the sinus during blunt dissection. Although the two-piece technique addresses this problem by allowing direct visualization of the sinus while dissecting the dura from the overlying bone before removing it, additional time is added to the procedure by requiring more bone cuts and further reconstruction when replacing the bone flap. Burr holes placed directly over the sinus avoid both of these issues, but this technique does not offer the same degree of control in exposure. An oscillating saw minimizes the complexity of reconstruction, but it is felt that this is largely a blind maneuver. Use of the 8MH17 bit and troughing craniotomy technique minimizes the risk of drilling into the sinus by allowing direct visualization of the venous sinus in addition to the dura on both sides. Additionally, the reconstruction is quite simple, and the cosmetic results are satisfactory. No bone cement is needed as the trough is thin enough (~2mm in diameter) to be unnoticeable by the patient postoperatively. In our series of 82 cases using this technique, no significant venous sinus injuries were encountered during surgery.

## Conclusions

Our findings with 82 patients show that a single-piece craniotomy using a trough technique is a safe, effective, and efficient option for dural venous sinus exposure with minimal reconstruction required at the close of the case. In our series, there were no intraoperative complications and no injuries to the dural venous sinuses using the troughing technique. While there are several methods that can be utilized when drilling over the dural venous sinuses, surgeons should consider the troughing method when preparing for removal of lesions near the falx or tentorium.
